# Supplementary material for: Identification of photoperiod-induced specific miRNAs in the adrenal glands of Sunite sheep (Ovis aries)
Source: Front Vet Sci. 2022 Jul 22;9:888207. doi: 10.3389/fvets.2022.888207 (PMC9354845; doi:10.3389/fvets.2022.888207)
Supplement: Supplementary file 1 [file Data_Sheet_1.ZIP › Supplementary file/miRDeep2 file.zip!/miRDeep2 file/Know_Structure/LP42A2/oar-mir-374a.pdf]

miRBase precursor : oar-mir-374a
Total read count : 22858
oar-miR-374a read count : 18524
remaining reads : 4334

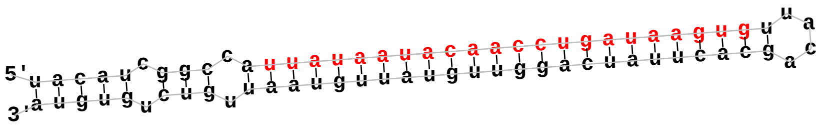

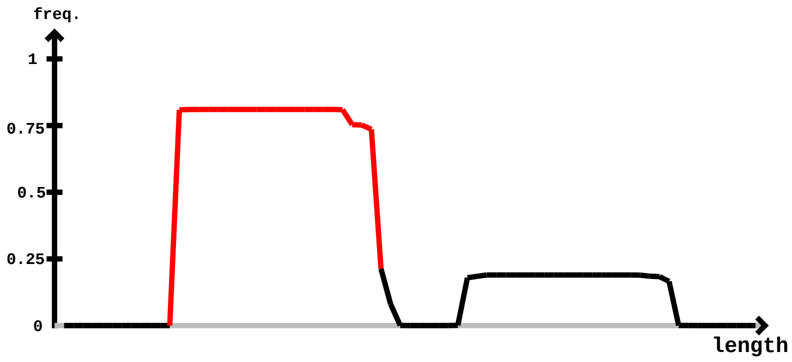


```
                   oar-miR-374a
5'- uacaucggccauuauaauacaaccugauaaguguuacagcacuuaucagguuguauuguaauugucugugua   -3'    exp
    (((((.(((.((((((((((((((((((((((((....)))))))))))))))))))))))).))).)))))    reads      mm         sample
    ...........uuauaauacUaccugau............................................    1          1          seq
    ...........uuauaauacaaccugau............................................    19         0          seq
    ...........uuauaauacaaccugGu............................................    1          1          seq
    ...........Auauaauacaaccugau............................................    1          1          seq
    ...........uuauaauacaaccugaA............................................    1          1          seq
    ...........uCauaauacaaccugaua...........................................    3          1          seq
    ...........uuauaauacaaccugauU...........................................    15         1          seq
    ...........uuauaauacaaccugGua...........................................    4          1          seq
    ...........uGauaauacaaccugaua...........................................    1          1          seq
    ...........uuaCaauacaaccugaua...........................................    19         1          seq
    ...........uuauaauacaaccugaua...........................................    1205       0          seq
    ...........Cuauaauacaaccugaua...........................................    11         1          seq
    ...........uuaAaauacaaccugaua...........................................    18         1          seq
    ...........uuauUauacaaccugaua...........................................    7          1          seq
    ...........uuaAaauacaaccugauaa..........................................    1          1          seq
    ...........uuauaauacaaccugauaa..........................................    30         0          seq
    ...........uuaCaauacaaccugauaa..........................................    1          1          seq
    ...........uuauaauacaaccugGuaag.........................................    1          1          seq
    ...........uuauaauacaaccugauaag.........................................    357        0          seq
    ...........uuaCaauacaaccugauaag.........................................    5          1          seq
    ...........uuaCaauacaaccugauaagu........................................    160        1          seq
    ...........uGauaauacaaccugauaagu........................................    1          1          seq
    ...........uuauaauacaaccugauaagu........................................    11782      0          seq
    ...........uGauaauacaaccugauaagug.......................................    1          1          seq
    ...........uuauaauacaaccugauaagug.......................................    2981       0          seq
    ...........uuaCaauacaaccugauaagug.......................................    44         1          seq
    ...........uuauaauacaaccugauaagugu......................................    1818       0          seq
    ...........uuauaauacaaccugauaaguguu.....................................    5          0          seq
    ............uauaauacaaccugaua...........................................    1          0          seq
    ............uauaauacaaccugauaagu........................................    9          0          seq
    ............uauaauacaaccugauaagug.......................................    1          0          seq
    ............uauaauacaaccugauaagugu......................................    10         0          seq
    ............uauaauacaaccugauaaguguu.....................................    4          0          seq
    .............auaauacaaccugauaagu........................................    2          0          seq
    ..............uaauacaaccugauaagugu......................................    4          0          seq
```

oar-miR-374a

uacaucggccauuauaauacaaccugauaaguguuacagcacuuaucagguuguauuguaauugucugugua			
.................uacaaccugauaaguguuacag.................................	1	0	seq
....acuuaucagguuguauugua............	1	0	seq
....acuuaucagguuguauuAua............	1	1	seq
....acuuaucagguuguauuguaa...........	3	0	seq
....acuuaucagguuguauuguaau..........	4	0	seq
....acuuaucagguuguauuguaauu.........	12	0	seq
....cuuaucagguuguauugu.............	10	0	seq
....cuuCucagguuguauugua............	1	1	seq
....cuuaucagguuguauugua............	91	0	seq
....cuuaucagguuAuauugua............	1	1	seq
....cuuaucagguuguauuAuaa...........	1	1	seq
....cuuaucagguuguauuguaa...........	26	0	seq
....cuuaucagguuguauuguaau..........	394	0	seq
....cuuaucagguuAuauuguaau..........	1	1	seq
....cuuaucagguuguauuguaauu.........	3546	0	seq
....cuuaucagguuAuauuguaauu.........	2	1	seq
....cuuaucagguuguauuguaauug........	3	0	seq
....uuaucagguuguauuguaau..........	7	0	seq
....uuaucagguuguauuguaauu.........	116	0	seq
....uaucagguuguauuguaa...........	1	0	seq
....uaucagguuguauuguaau..........	6	0	seq
....uaucagguuguauuguaauu.........	102	0	seq
....uaucagguuguauuguaauugu.......	4	0	seq
